# UAV Atmosphere Sounding for Rocket Launch Support

**DOI:** 10.3390/s23249639

**Published:** 2023-12-05

**Authors:** Karol Piotr Bęben, Tomasz Noga, Dawid Cieśliński, Dawid Kulpa, Marcin Ryszard Spiralski

**Affiliations:** 1 Remote Sensing Department, Unmanned Technologies Center, Łukasiewicz Research Network—Institute of Aviation, 02-256 Warsaw, Poland; dawid.kulpa@ilot.lukasiewicz.gov.pl; 2 Rocket Technologies Department, Space Technologies Center, Łukasiewicz Research Network—Institute of Aviation, 02-256 Warsaw, Poland; tomasz.noga@ilot.lukasiewicz.gov.pl (T.N.);

**Keywords:** atmosphere sounding, unmanned aerial system, unmanned aerial vehicle, real-time, rocket launch, sensors, wind measurement

## Abstract

One of the crucial branches of activity at the Łukasiewicz Research Network—Institute of Aviation is developing a suborbital rocket vehicle capable of launching small payloads beyond the Earth’s atmosphere, reaching over 100 km in altitude. Ensuring safety is a primary concern, particularly given the finite flight zone and impact area. Crucial to safety analysis is the wind profile, especially in the very first seconds of a flight, when rocket velocity is of the same order as the wind speed. Traditional near-ground wind data sources, ranging from wind towers to numerical models of the atmosphere, have limitations. Wind towers are costly and unfeasible at many test ranges used for launches, while numerical modeling may not reflect the specific ground profile near the launcher due to their large cell size (2 to +10 km). Meteorological balloons are not favorable for such measurements as they aim to provide the launch operator with a wind profile at high altitudes, and are launched only 1–2 times per flight attempt. Our study sought to prototype a wind measurement system designed to acquire near-ground wind profile data. It focuses on measuring wind direction and speed at near-ground altitudes with higher flight frequency, offering data on demand shortly before launch to help ensure safety. This atmosphere sounding system consists of an Unmanned Aerial Vehicle (UAV) equipped with an onboard ultrasonic wind sensor. Some reports in the literature have discussed the possibility of using UAV-borne anemometers, but the topic of measurement errors introduced by placing the anemometer onboard an UAV remains under studied. Limited research in this area underlines the need for experimental validation of design choices–for specific types of UAVs, anemometers, and mounting. This paper presents a literature review, a detailed overview of the prototyped system, and flight test results in both natural (outdoor) and controlled (indoor, no wind) conditions. Data from the UAV system’s anemometer was benchmarked against a stationary reference weather station, in order to examine the influence of the UAV’s rotor on the anemometer readings. Our findings show a wind speed Root Mean Square Error (RMSE) of 5 m/s and a directional RMSE of below 5.3° (both averaged for 1 min). The results were also compared with similar UAV-based wind measurements. The prototyped system was successfully used in a suborbital rocket launch campaign, thus demonstrating the feasibility of integrating UAVs with dedicated sensors for performing regular meteorological measurements in automatic mode.

## 1. Introduction and Literature Review

In recent years, Unmanned Aerial Systems (UAVs) have emerged as a valuable tool in various industries, notably including atmospheric research and weather forecasting [1]. These autonomous unmanned systems provide researchers with an alternative method of measuring wind speed and direction in areas that are difficult to access or pose a risk to a human operator. The traditional methods of measuring wind speed and direction involve using fixed-wing or manned aircraft, whereas small UAVs have been shown to be more cost-effective and flexible in meteorological data acquisition near the surface [2].

Unmanned aerial vehicles can, therefore, be expected to come into use for taking meteorological measurements in all sectors where near-surface troposphere measurements are essential–including aerospace, search and rescue, and sports competitions such as gliding or ski jumping. This capability has already been demonstrated in such applications as fire monitoring [3] and wind turbine wake mapping [4].

The use of UAVs equipped with advanced wind sensors is particularly promising in terms of providing real-time and accurate wind data during rocket launches. Accurate wind profiling is crucial for ensuring a high level of safety and success ratio for such launches, as wind conditions can greatly affect a rocket’s trajectory and stability. Using UAVs equipped with wind sensors, meteorologists and launch operators can gather real-time data on wind speed and direction at different altitudes.

UAVs, equipped with ultrasonic anemometers, are particularly adept at this, offering the ability to hover and accurately collect detailed data on wind velocity and direction in three directions. While fixed-wing UAVs with pitot-based wind sensors have been used for atmospheric research for the past two decades, rotary-wing aircraft offer superiorities such as the ability to hold their position mid-air for extended periods of time, allowing for more detailed and comprehensive wind measurements [5].

Research has explored various methods for measuring wind using UAVs. Studies have demonstrated methods including the use of weather observation towers for reference, comparing direct anemometer measurements on UAVs with independent wind data, and developing wind vector formulas based on UAV navigation data. These studies highlight the influence of UAV rotors on anemometer readings and the challenges in aligning UAV and anemometer data. Some researchers have also explored indirect measurement methods, correlating UAV inclination with wind speed and direction, and testing these relationships in controlled environments.

In the study by Shimura et al. [6], a UAV maintained a constant altitude of either 40 or 55 m above ground level for a duration of 15 min to collect data, which was then compared to weather tower observations at two different heights. The ultrasonic anemometer aboard the UAV measured wind at 1-s intervals, contrasting with the estimation period for the tower’s anemometer (averaging more than 1 min). Data analysis showed a 0.5 m/s wind speed bias and −9° bias in direction. The Root Mean Square Error (RMSE) values for wind speed and wind direction were determined as 0.6 m/s and 12°, respectively. The study also showed that wind speeds went up by about 0.5 m/s while the device was hovering, which could be explained by the rotors affecting the anemometer, whereas UAV tilting was found to have minimal impact.

Palomaki et al. [7] compared two UAV-based wind measurement methods: directly using an anemometer, and indirectly derived from the UAV’s motion. The anemometer onboard the UAV and a 10-m tower were used for the direct approach to measure wind vectors. In the indirect method, wind vectors were found by using data gathered from pitch, roll and yaw angles. The data from the UAV, recorded at 10 Hz, was juxtaposed with 1 Hz recordings from 2D sonic anemometers on 3 masts. The direct method yielded worse results in terms of wind speed but better results for wind direction. The researchers noticed that there were times when the discrepancy in wind speeds between the three towers was more than 1 m/s.

Neumann and Bartholmai [8], in turn, developed wind vector formulas from UAV navigation sensor data. Their approach, validated through wind tunnel and in-flight tests, modeled the flow speed as a function of the UAV’s tilt angle. The hovering test was performed 2–5 m away from the anemometer to ensure that the airflow generated by the micro-UAV rotors did not interfere with the anemometer. Data from the UAV and anemometer were collected at various frequencies for 20 min. The RMSE for speed and direction was affected by data processing. Potential differences include assuming a steady wind field, using different measuring points, modifying the distance between the micro-UAV and the anemometer, and time differences due to GPS and IMU data were not always in sync. Position holding accuracy was calculated to be 1.17 m. Zhengnong Li et al. [9] used a similar method, mounting an anemometer on a UAV, conducting wind tunnel tests as well as comparing measurements from the UAV and the wind tower’s anemometer. Both direct measurements and comparisons of the power spectra of those measurements showed that the UAV and wind tower were in good agreement.

Brosy et al. [10] used an indirect measurement method, correlating UAV tilt angles to Total Angle of Sight (TAS) during flight experiments. The UAV’s tilt angle was calculated with precision greater than 0.1° using pitch and roll angles. Racetrack flights were used to test the regression function when the speed of the wind during the test was reported as less than 1 m/s. The UAV hovered around 5 m above the ground, and an ultrasonic anemometer was used to verify the accuracy of the approach. After 5 min of hovering, the UAV-derived wind direction had a standard deviation of ±11.1° with the wind speed standard deviation of ±0.7 m/s. The differences in readings from the UAV and the tower over the course of 5 min were 7.7° and 0.3 m/s, respectively. The UAV could record changes in both the speed and direction of the wind. However, the UAV’s large size compared with the sonic anemometer’s measurement path meant that it could not record the full range of wind speeds.

Tianhao Hou et al. [11] adopted a novel approach, outfitting a quadrocopter UAV with four custom-made anemometers, one for each rotor arm. This method differed from typical off-the-shelf anemometers, yielding slightly better RMSE accuracy than reported elsewhere. Prudden et al. [12] described the development of a sensor suite for atmospheric boundary layer measurements with a small multirotor unmanned aerial system (UAS), including anemometers, thermometers, pressure sensors, and humidity sensors. The sensor suite was integrated on the sides of a multirotor UAS DJI Matrice M600 and was used to collect data in a variety of atmospheric conditions.

Methods based on UAV dynamics require knowledge of flow speed as a function of tilt angles, while methods utilizing an anemometer require modifications to the UAV. Methods utilizing UAV dynamics can be used on any UAV as long as the relation of the UAV’s dynamics to wind conditions is known or can be derived. These flight dynamics are recorded within onboard logs, which can be then read and transformed into wind speed estimations. The literature presented above concerns multi-rotor UAVs only–there are also methods using fixed-wing UAVs, i.e., using a set of probes [12] or a “wind-arc” method developed in the Embry-Riddle Aeronautical University [13], but these lie beyond the scope of this paper. Performance comparisons between a quadrocopter and a fixed-wing UAV with a lidar used for reference were reported by Bronz et al. [14], and a fixed-wing system UAV system was developed by Witte et al. [15]. There is also a series of articles addressing small multi-rotor UAVs: discussing using sUAS to measure wind [16] or turbulence [17] and using drone swarms to measure wind [18]. But instead of focusing on the system design, these studies pay more attention to how to use the data that the UAV gives them and how to obtain information out of it using algorithms. Innovative approaches in wind sensor development for UAVs are also being tested [19].

This literature review has discussed in detail five experiments in which the wind vector was measured using a UAV–three utilizing an anemometer onboard the UAV, and three computing the wind vector using the UAV’s tilt angle. The RMSE reported for wind speed ranges from 0.27 m/s (with bias removed) to 1.09 m/s, while the RMSE reported for wind direction is from 7.7° up to 56°. All these papers performed validation by comparing measurements from the UAV to measurements from the anemometer on a tower or a mast–indicating that carrying out a UAV-based wind measurement experiment essentially requires comparison against some reference measurements. Two measurement methods have been discussed in the literature–using an anemometer attached to a UAV and measurement of UAV dynamics. The first method has the advantage of being easier to develop regardless of the UAV vehicle used.

This paper presents a multi-rotor UAV wind vector measurement system that was developed and validated in-house at the Łukasiewicz Research Network—Institute of Aviation. The system is designed to provide wind profile measurements for an ILR-33 AMBER 2K suborbital rocket launch campaign. This paper is structured as follows. Section 2 presents the materials and methods used to develop and validate the system. The detailed UAV design, wind measurement methods used, software and communication protocols and experimental set-ups are outlined and justified. Section 3 presents the results from test campaigns. Section 4 discusses these results and describes how UAV systems verified by the tests can be used to enhance meteorological monitoring for a suborbital rocket launch campaign. Section 5 presents conclusions and proposes future work.

One of the applications that potentially stand to benefit from the presented solution is launch vehicles. Traditionally, wind profile measurements for such applications have relied on meteorological balloons [20] or wind towers with meteorological stations mounted at various heights. The disadvantages of the former solution mainly include the minimal number of measurements, wind drift of the balloon potentially affecting balloon data, and its unsuitability for prolonged, higher-resolution monitoring over an extended period during the day for a given location. The latter approach, involving measurement towers, has the disadvantage of entailing high costs of construction, maintenance, and logistics, including security considerations. Moreover, towers have height limitations and may not be feasible at particular launch locations, especially in the case of countries lacking a well-established suborbital rocket industry. In contrast, UAV-based measurements offer a cost-effective solution that can reach greater altitudes. Their high flexibility allows the wind profile to be measured at chosen locations, which can be very close to the launcher.

The primary objective of this system is to gather reliable data that can serve as crucial input for simulations and numerical models, aiding in the prediction of wind behavior. The results derived from these analyses play an integral role in the decision-making process related to rocket launches. By utilizing our UAV-based wind measurement system, launch operators can make more informed decisions about the timing and safety of their missions, ultimately improving the success rate of suborbital rocket launches.

## 2. Materials and Methods

**Figure 1:**
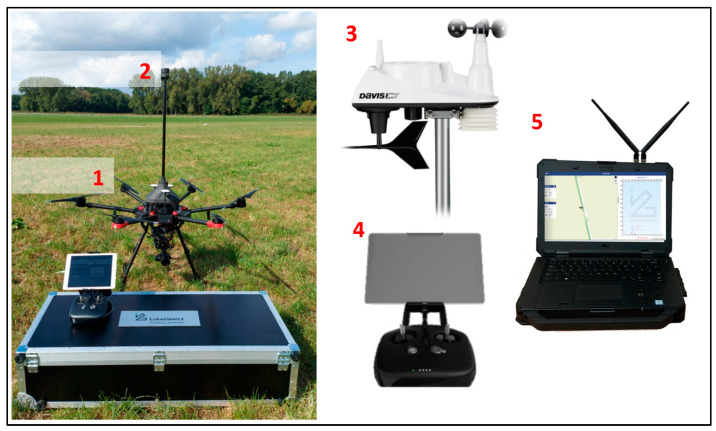
UAV sounding system devices used during the test. Credit: Łukasiewicz Research Network—Institute of Aviation.

**Figure 2:**
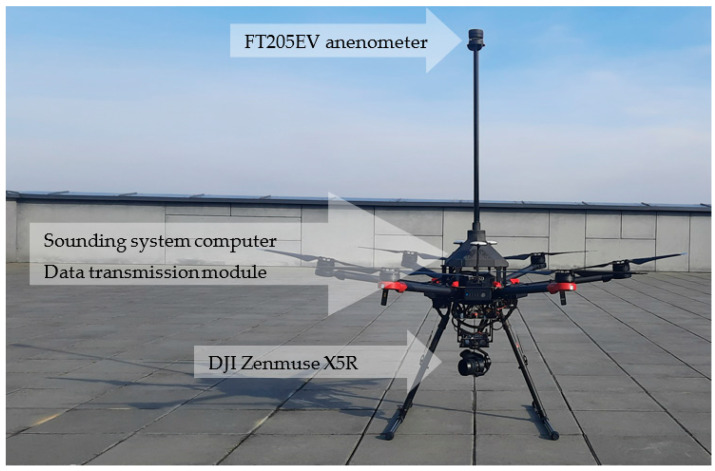
DJI Matrice 600 Pro with weather sounding system installed. Credit: Łukasiewicz Research Network—Institute of Aviation.

This section first presents the prototype of the wind measurement system, describing the details of the measurement component (anemometer) and flight component (UAV with software) (Figure 1 and Figure 2). Subsequent paragraphs describe the UAV mission planning and automation processes required for the flight tests presented in the next section. The concluding paragraph highlights the potential limitations of our study and the methods applied.

Figure 1 presents the prototype system, consisting of (1) an unmanned aerial vehicle (UAV), with a wind speed and direction measuring device (anemometer) attached, (2) a reference weather station measuring real-time wind speed and direction, (3) algorithms executing a user-defined flight sequence, and (4) a ground station with an algorithm (5) comparing wind speed and direction readings from the measuring device on the UAV with the meteorological station placed on the mast.

For the wind measurement component, we employed the FT 205 EV (FT Technologies Ltd., Sunbury, UK) digital ultrasonic anemometer. This anemometer was securely fastened to the hexacopter structure of the DJI Matrice 600 Pro Unmanned Aerial Vehicle (DJI, Shenzhen, China), as depicted in Figure 2. To minimize disturbances arising from rotor-generated airflow, the sensor-anemometer was strategically positioned at a safe horizontal and vertical distance from the rotors and propellers. This positioning maintained alignment with the carrier platform’s axis and ensured parallel orientation with the north direction.

The anemometer frame was fabricated using the Multi Jet Fusion 3D printing method, incorporating composite technology with carbon fiber for the mast and center plate. Note, that Fused Deposition Modeling (FDM) and Selective Laser Sintering (SLS) 3D printing technologies are not recommended for complex or extensive in-volume models [21].

During the test campaign, we employed the Davis Vantage Vue reference meteorological station. Equipped with a mast, this station could be placed at altitudes of up to 12 m above ground level. In terms of wind measurement capabilities, it can record wind speeds up to 89 m/s with an accuracy of 1 m/s or 5%, depending on which of those values is higher (in the cases analyzed here, it is ±1 m/s), and wind direction with an accuracy of ±3 degrees. The station provides 1-min-average results, saved and analyzed in postprocessing [22].

**Figure 3:**
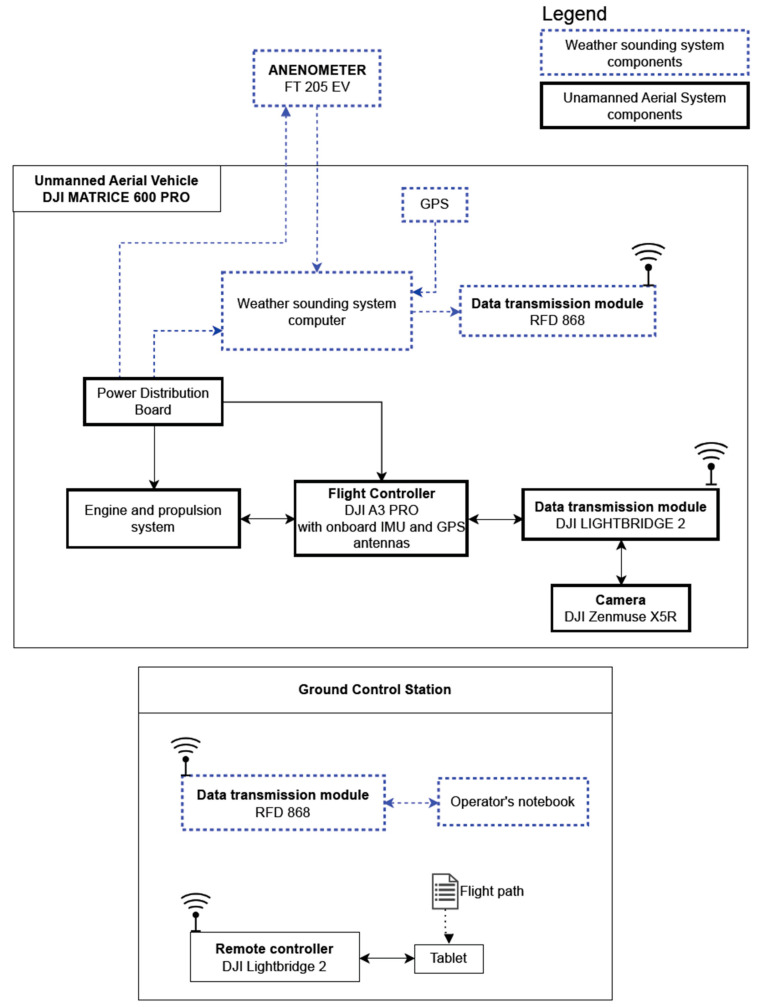
Main components diagram of Unmanned Aerial Vehicle with weather sounding system. Credit: Łukasiewicz Research Network—Institute of Aviation.

Figure 3 presents a schematic overview of the connections between the various components of the atmospheric sounding system. The FT205EV sensor was connected to a local onboard mini-PC, which was also integrated into the carrier platform. The sensor communicated with the computer via a serial port and a USB-TTL/UART converter.

The on-board computer ran on the MS Windows operating system. It was equipped with a GPS receiver and proprietary software (EmbMeteoApp v1.0, (EMApp)) apable of collecting information from the installed sensors in NMEA 0183 format. These data were decoded and transmitted as NMEA 0183 to the RFD 868 MHz Wireless Communication Module. The FT Technologies FT205EV ultrasonic anemometer measured wind speed and direction, while the GPS module was additionally used to determine the location at the time of measurement.

The Ground Control Station runs on the Windows operating system with proprietary software EMApp, receiving data frames via an RFD868 radio modem. The software at the ground station processed the data frames into a graphical visualization of wind speed and direction, which were then saved to a text file.

The entire weather-sounding system onboard the UAV was powered by the onboard power source that is supplied with the DJI Matrice 600 Pro carrier platform. The power supplies for the mini-PC computer, the RFD868MHz radio modem and the sensor were provided by adjustable converters.

The flight sequence comprises a series of maneuvers, starting with ascending to pre-determined altitudes. At each designated altitude (waypoint), the UAV maintains its position for a predetermined measurement duration before ascending to the next designated waypoint. This process continues until the maximum altitude is achieved. Subsequently, the UAV begins its descent, retracing its path and conducting measurements at the same waypoints as during the ascent phase. This cycle repeats until the UAV safely lands. Notably, both takeoff and landing are executed in automatic mode. The measurement procedures utilize both the UAV-mounted measuring device and a device on a stationary mast in an area devoid of terrain obstacles. Table 1 lists the detailed parameters of the designed flight mission.

**Figure 4:**
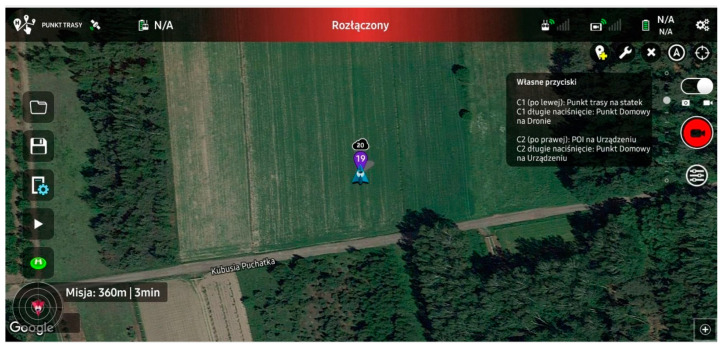
Waypoints module on the Litchi software. Credit: Łukasiewicz Research Network—Institute of Aviation.

Flight path planning was performed using Litchi (VC Technology, London, UK) software, Figure 4. This software is also used to control the UAV during the mission. Table 1 contains the critical parameters for the mission flight plan. While regulations for normal UAV operation in the European Union limit the altitude to 120 m AGL (Above Ground Level), the system can fly to an altitude of up to 500 m AGL. To perform a UAV flight above 120m AGL, permission must be obtained from the Civil Aviation Authority (CAA, Warsaw, Poland). Such permission is issued based on the UAV operation’s risk analysis using the SORA (Specific Operations Risk Assessment) methodology. Regular UAV flights up to 120 m can be performed in order to confirm the reliability of the system, which is required to obtain the aforementioned permission for higher flights [23].

To demonstrate the operational capabilities of the system, a series of staged tests were conducted, progressively increasing the complexity of both flight parameters and flight conditions. Testing began with controlled indoor assessments of the system, conducted in a windless environment. Subsequently, outdoor field tests were performed under indeterministic conditions, including wind gusts. Throughout each stage, the acquired data were meticulously compared against that of a reference ground meteorological station.

As our research team became more familiar with the proposed system and the testing plan, several noteworthy limitations became apparent to us. We primarily relied on the ground station (Davis) as the reference source, accompanied by a certificate of conformance validating the device’s accuracy as provided by the supplier. However, the station had been utilized several times in real missions, such as rocket flight attempts, and there was no available information regarding its potential degradation over time. Implementing a calibration procedure, possibly involving wind tunnel testing, could have mitigated uncertainties stemming from the reference system. Regrettably, such calibration efforts exceeded the available funding for the prototype development project detailed in this paper.

In order to eliminate potential uncertainties originating from interference between the measurement units, an indoor test campaign was conducted, including a test for determining the minimum distance at which any interference occurred (between rotors vs. ground reference stations). The outdoor tests were executed at relatively low wind speeds <7 m/s. According to the UAV manual, it is capable of withstanding winds up to 8 m/s. Therefore, any higher wind values would require an additional algorithm to account for possible drift of the UAV due to strong winds (such considerations are not addressed in this paper). Finally, the UAV aerodynamics configuration remained fixed throughout the testing. No modifications were made to the rotors to search for any potential source of thrust misalignment-such as determining which rotors (blades) generated the least or most force. The initial test results, where the UAV was anchored to the ground UAV and powered up, did reveal an initial error attributed to misalignment. This error primarily affects the measurement of direction rather than the magnitude of velocity, but the exact root cause (location) of the misalignment was not investigated.

## 3. Test Campaign–Design and Results

Several tests were performed to estimate how well the UAV-based atmospheric sounding system operates and what errors are encountered. These tests followed methodologies similar to those used in previous studies, as described in the literature review, primarily in the sense that readings made by the anemometer onboard the UAV were compared to those taken by a reference weather station. Section 3.1, Section 3.2 and Section 3.3 describe the successive tests and analyze their results; Section 3.4 describes the first application of the UAV sounding system–supporting a sounding rocket launch campaign.

### 3.1. Impact of UAV Rotors on a Reference Weather Station

**Figure 5:**
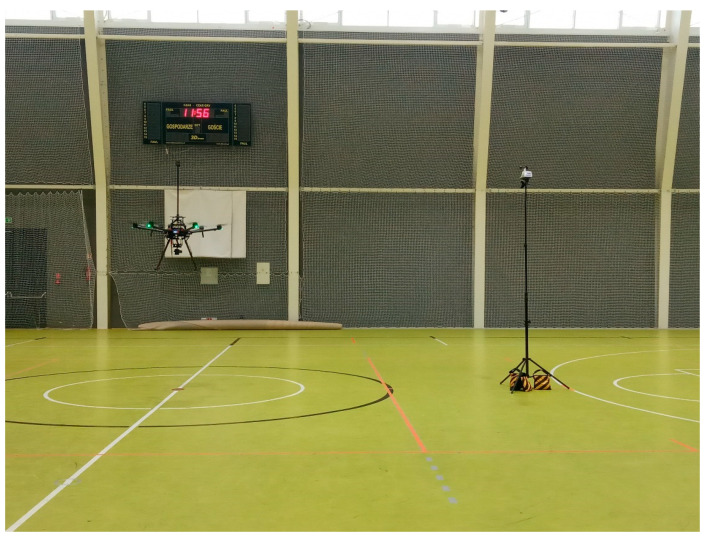
UAV and meteorological station during the hovering test in the sports hall. Credit: Łukasiewicz Research Network—Institute of Aviation.

The first aspect tested was to verify if the UAV rotors would influence the readings of a nearby reference weather station. Both the reference weather station and the UAV were placed indoors, in a sports hall with no-wind conditions. Both stationary and hovering tests were performed. The UAV’s rotors operated at different relative altitudes and horizontal distances from the weather station, with 5 m being the closest (Figure 5). The tests confirmed that none of the configurations influenced the reference station’s wind measurements, provided that both the UAV and reference stations were positioned at altitudes over 2.5 m (avoiding any rotor downwash effects from the ground surface).

### 3.2. Impact of UAV Rotors on the Onboard Anemometer Readings

**Figure 6:**
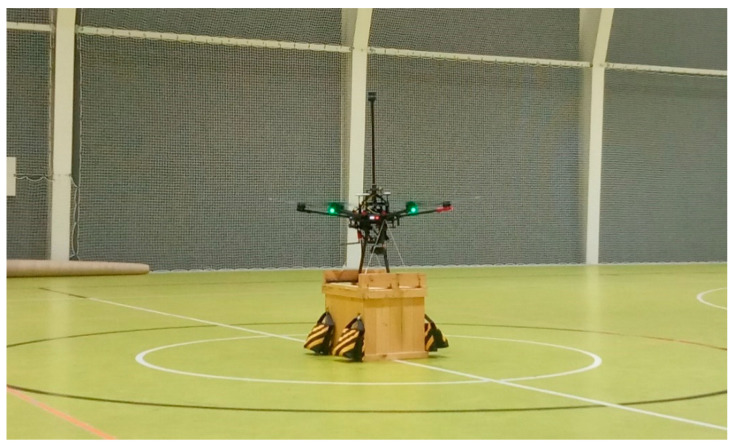
Stationary rotor-bias test. Credit: Łukasiewicz Research Network—Institute of Aviation.

Another critical aspect of the test campaign was assessing how the UAV’s rotors impacted its onboard anemometer readings, which is a known source of measurement error [24,25]. These tests were also conducted in the controlled, no-wind environment of the sports hall. Initially, the UAV was secured to the ground (Figure 6), and six trials were performed with the rotors operating at varying RPMs. The results, presented in Table 2, indicated that low RPMs caused the anemometer to register wind speeds around 0.3 m/s, whereas high RPMs resulted in readings close to 1 m/s. The average wind direction recorded in both test conditions was approximately 170°.

### 3.3. Field Tests

**Figure 7:**
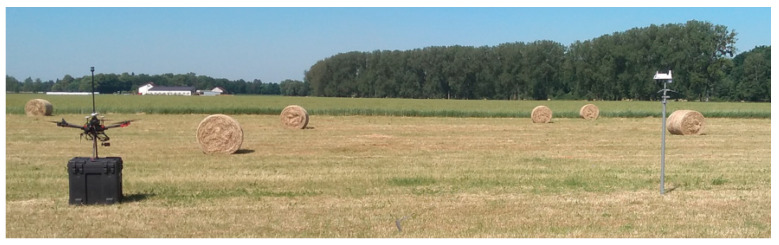
UAV and meteorological station during the stationary field test. Credit: Łukasiewicz Research Network—Institute of Aviation.

A field test was performed to compare results from the UAV-mounted anemometer with results from the meteorological station on a mast. These tests had two phases: a stationary test and a hovering test. In the stationary test, the UAV had its rotors powered off, wind gusts only) and other sources of error or disturbance during the UAV flight (anemometers comparison). The hovering test, on the other hand, allowed comparison of a UAV in flight conditions. During the first test, the UAV was stationary (Figure 7), with rotors off, and was positioned so that its anemometer was at the same height as the meteorological station on a mast (~2.3 m). This setup was intended to compare readings without the influence of the UAV’s rotors or other potential disturbances. The meteorological station, lacing a magnetometer or other sensor, was manually oriented to the North before measurements started, using a compass while taking magnetic declination into account. The overall error of this positioning was estimated to be 10°. The UAV anemometer was positioned 10 m south of the meteorological station, in line with the eastward wind direction at that time, it was desired to have a line between sensors perpendicular to the wind vector to ensure wind gusts reach both sensors simultaneously. The distance of 10 m was chosen for consistency with the hovering test. Time in the UAV logging station and on the meteorological station logging system were synchronized manually, with an error of no more than 1 s. Data were gathered for 30 min.

**Figure 8:**
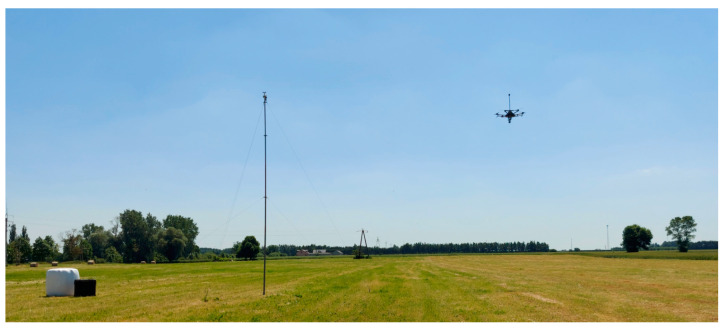
UAV and meteorological station during the hovering field test. Credit: Łukasiewicz Research Network—Institute of Aviation.

For the hovering test, the meteorological station’s mast was elongated and positioned ~8 m above the ground level. The UAV was then launched and positioned 10 m from the meteorological station. Then, it was set to a GNSS hold mode while maintaining constant height, ensuring the anemometer was 8 m above the ground level (Figure 8).

Five hovering tests were conducted, lasting from 13 to 19 min each. The azimuth from the UAV to the station was set prior to each test to ensure that the wind direction would not be parallel to it, to minimize the time between the arrival of wind gusts to the sensors.

**Figure 9:**
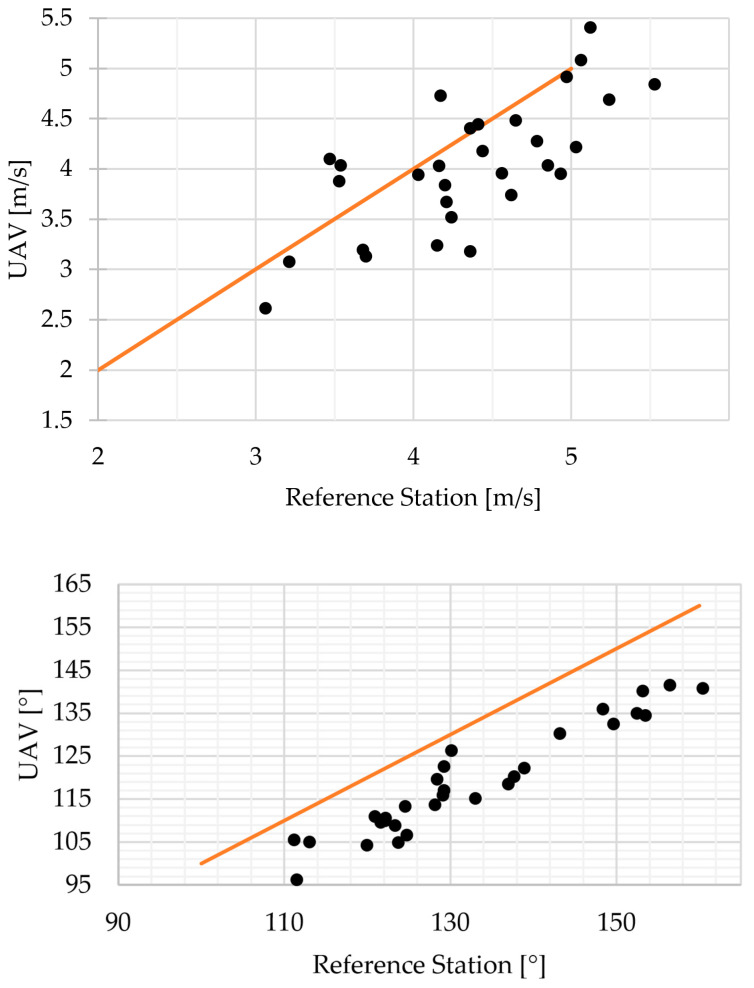
Stationary test results–velocities (**top**) and directions (**bottom**).

The results of the stationary test are plotted in Figure 9.

The results from the stationary test showed a velocity bias between the UAV and the reference station of 0.69 m/s with an RMSE of 0.47 m/s. The mean difference between directions was −13.49°, with RMSE of 4.13°. Recall that the orientation of the reference station could not be set precisely and an error of +/− 10° for the reference station orientation was to be expected.

**Figure 10:**
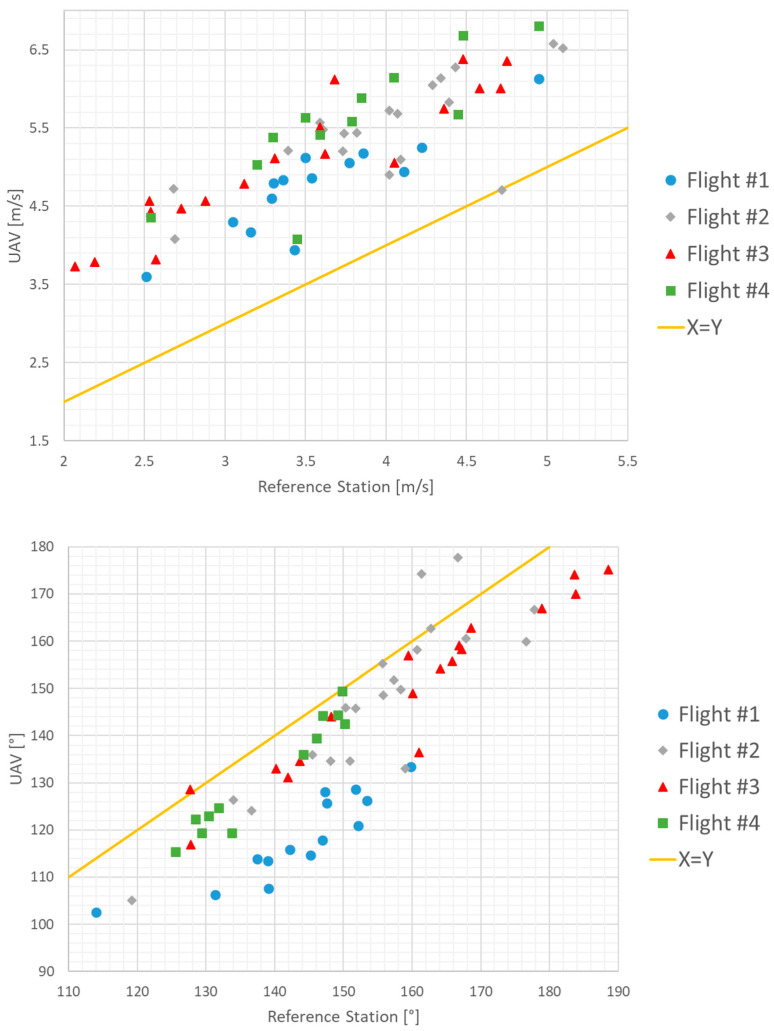
Hovering test results–velocities (**top**) and directions (**bottom**).

The results from the four hovering tests are plotted in Figure 10 and their statistics is presented on Table 3. The overall bias calculated using data from all four flights is 1.5 m/s, and the corresponding RMSE is 0.48 m/s. The overall direction bias is −12.52° and the corresponding RMSE is 5.33°. These findings were compared with other UAV-based wind measurement methods found in the literature, as given in Table 4.

### 3.4. The First Application–Sounding Rocket Launch Campaign

**Figure 11:**
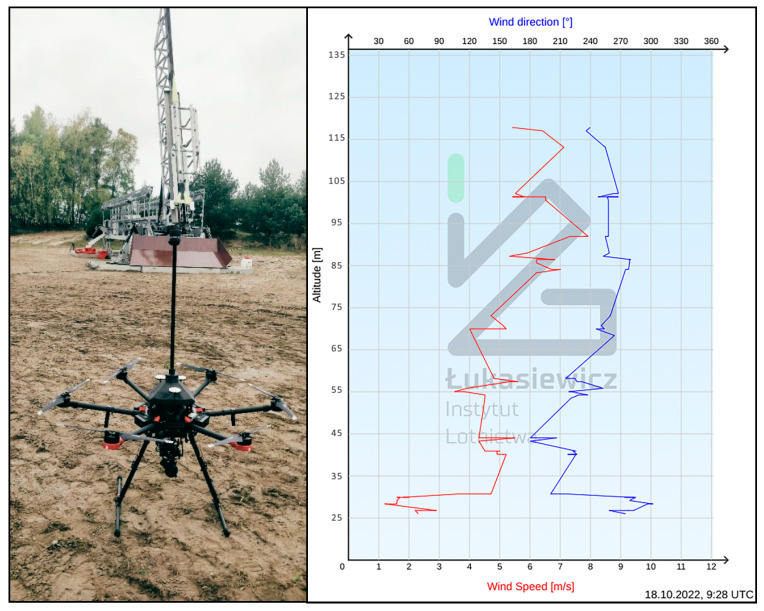
UAV with an anemometer and the ILR-33 AMBER 2K rocket on the launch pad (in the rear) prior to the test flight. Atmospheric sounding results up to a height of 120 m AGL at the Air Force Training Center Ustka presented in the chart on the right.

The first practical application of the system took place during the flight campaign of the ILR-33 AMBER 2K rocket [26,27,28]. The UAV anemometer system offers a mobile and low-cost alternative to the wind tower (with the FT 205 EV anemometer costing about $4000 USD). The prototype system described in this paper was successfully deployed on 18 October 2022 (Figure 11). The system performed well, providing mission planners with wind data for altitudes up to 120 m. Such information is crucial for flight analysis, enhancing the accuracy of simulations and improving overall flight safety.

## 4. Discussion

Utilizing UAV-based atmospheric sounding systems holds the potential to significantly enhance safety in operations sensitive to wind variability, like airshows. By capturing turbulence in air motion or gusts above the terrain, they make it possible to issue appropriate warnings to interested parties (i.e., pilots at airshows). Additionally, high-resolution data from UAV soundings can refine numerical weather models, especially for small areas (of the order of several km^2^), by providing detailed wind parameters both vertically and horizontally. This is particularly beneficial for sectors like the military, wind energy, and the construction of radio masts and towers.

Our study’s findings (presented in Table 4) show that while the wind speed bias of the UAV system is higher than reported for other designs (about 1.5 m/s versus 0.5 m/s), the RMSE for wind speed is actually lower (around 0.5 m/s compared to 0.6–1.2 m/s). However, once identified, the bias can be corrected for and does not significantly impact accuracy. The larger bias might be due to the UAV’s rotor influence, possibly because of its heavier take-off weight (over 10 kg) compared to other models used in the literature. The direction bias and RMSE are consistent with other studies, with the direction RMSE being lower likely due to a longer averaging time scale (1 min).

To mitigate bias from UAV rotor effects on anemometer measurements, the anemometer was mounted on a specially designed arm. This design was informed by the approach used in [25], facilitating a similar system configuration. Alternative mounting strategies were explored, such as the side-mounted mast approach suggested in [7], akin to the designs in refs. [11,29]. However, the top-mounted mast design, as in refs. [6,25], where a mast with an FT205 anemometer was placed atop the drone, was ultimately chosen. This decision was influenced by various factors, including drone design. The referenced works used different drone sizes, ranging from smaller models in refs. [7,29] to larger ones like the DJI Matrice 200 series in [6] and a high-capacity hexacopter in [25]. The last two models mentioned are more akin to the drone used in this research.

Empirical data from this study and literature review affirm the viability of both anemometer mounting approaches on UAVs for wind measurement. Other potential error sources, such as inaccuracies in the anemometer or UAV navigation errors leading to drift, were also considered, as discussed in the Materials and Methods section. The reference station used in the study, while being a potential source of measurement error, was found not to be affected by UAV-induced vortices (downwash). Nonetheless, it is clear that the uncertainty inherent in UAV-based measurements, when compared against a reference station, will inherently be greater than the uncertainty of the reference station itself. Additionally, factors like uneven wind fields and gusts also impact the study’s results.

Obtaining real-time results was critical for the application of collecting data for rocket mission modeling. While dataloggers, as utilized in [25], may be sufficient for other applications, for our purposes data were needed in near real-time for inclusion in the rocket trajectory simulations. For a UAV descending from an altitude on the order of 500 m AMSL, assuming 5 m/s, the descent time is about 100 s. The extra time to retrieve data from the datalogger could extend the overall duration of data processing procedures to about 5 min. It should also be pointed out that other prelaunch procedures may also be dependent on the measurement results. Often, these activities are constrained by narrow time windows, necessitating swift launch execution with a specific configuration. Therefore, the approach outlined in this study, focusing on real-time data acquisition, is particularly well suited to the applications being considered.

The UAV weather sounding system’s capabilities can also be extended with a system for measuring various air parameters. This could encompass particulate matter sensors, e.g., PM2 and PM10, and the integration of CO_2_ measurements, as exemplified in [25]. Additionally, the system could incorporate temperature sensors (as in refs. [19,30]) and humidity sensors [12]. However, this would extend the use of the system to respond to somewhat different research problems, serving a wider range of purposes than the main focus of the present study. The data gathered from these sensors could be invaluable in diverse areas, such as predicting the spread of fires [12], studying the impact of terrain obstacles on air traffic, aiding in glider competitions, and analyzing air quality issues like smog. They could also be crucial in studying meteorological phenomena of a local character, such as turbulent wind movement, urban heat islands, or atmospheric inversions.

## 5. Conclusions

The results from the flight test campaigns verified the feasibility of using a UAV equipped with an onboard anemometer for atmospheric sounding. The system adhered to its primary design assumptions, and analysis of the collected data indicated that a hovering UAV does influence the onboard anemometer readings. However, these errors were largely accounted for during post-processing. The identified wind speed and direction biases were 1.5 m/s and −12.33°, respectively, with RMSE values of 0.5 m/s and 5.33°, which are within expected ranges for such devices.

The system’s successful application in a rocket flight campaign (for the first time) demonstrated its potential for enhancing mission safety. This approach is particularly beneficial for smaller launch sites (where horizontal zones are not as extensive as at the largest spaceports, like Cape Canaveral or Guyana), offering improved trajectory predictions and safer operations compared to the traditional weather ballooning methods typically used at smaller sites. UAV-based wind profiling can complement balloon sounding systems while providing more detailed temporal and vertical resolution in meteorological measurements.

In terms of adaptability, the system proposed herein will be easily compatible with a range of popular UAV models on the market. The process of assigning mandatory classes for UAVs, stemming from Commission Delegated Regulation (EU) 2019/945, may be more complicated for less advanced UAVs than products from DJI, AUTEL, Yuneec. Therefore, we propose a solution that will be adaptable to a drone that will be given a C0–C6 class. This will make it a more economically viable option for meteorological measurements, especially for institutions with budget constraints.

Future developments should focus on enabling automatic flight missions up to 500 m AGL and refining data analysis. Adapting the system for various popular UAV models, in line with the mandatory certification system in place starting in 2024, will align with the World Meteorological Organization’s requirements for meteorological measurement systems. Further research could include wind tunnel testing and integrating wind estimation calculations from UAV navigation data to enhance wind vector accuracy.

## Figures and Tables

**Table 1:** Main parameters of UAV weather sounding system flight plan.

Geographical Coordinates (WGS-84) of all Waypoints the Same			
Flight Phase	Parameter	Value	Unit
Climbing flight	First Waypoint AGL (Above Ground Level) Altitude	20	m
	Vertical distances between waypoints	20	m
	Last Waypoint Altitude	120	m
	Action on every Waypoint	Hover	n/a
	UAV Hover duration	5	s
	UAV Vertical Speed	5	m/s
	Flight path curve size	0	m
	Azimuth	0	°
	POI (Point of Interest)	None	n/a
	Gimbal Pitch Angle	Disabled	n/a
Descending flight	First Waypoint AGL (Above Ground Level) Altitude	120	m
	Vertical distances between waypoints	20	m
	Last Waypoint Altitude	20	m
	Action on every Waypoint	Hover	n/a
	UAV Hover duration	5	s
	UAV Vertical Speed	5	m/s
	Curve size	0	m
	Azimuth	0	°
	POI (Point of Interest)	None	n/a
	Gimbal Pitch Angle	Disabled	n/a

**Table 2:** Stationary rotor-bias test results.

	Velocity [m/s]			
	Low RPM		High RPM	
Test Number	Average	Median	Average	Median
1	0.27	0.3	0.55	0.6
2	0.29	0.3	1.09	1.1
3	0.41	0.4	1.18	1.2
1, 2, 3	0.33	0.30	0.96	1.10

**Table 3:** Field test results.

Test Case	Velocity [m/s]		Direction [°]	
	Bias	RMSE	Bias	RMSE
Stationary	0.69	0.47	−13.49	4.14
Flight #1	1.20	0.28	−25.14	5.14
Flight #2	1.34	0.90	−7.29	8.76
Flight #3	1.66	0.32	−9.45	5.18
Flight #4	1.79	0.43	−7.24	3.45
Overall Flight #1–4	1.497	0.48	−12.2	5.33

**Table 4:** Summary of UAV-based wind measurements.

UAV name	SPIDER CS6	DJI Flame Wheel F550	Quanum Nova	AirRobot AR100-B	DJI F550 Flame Wheel	DJI F450	DJI Matrice 600Pro
Wind sensing method	ultrasonic anemometer (FT702);	ultrasonic anemometer (Decagon Devices DS-2)	IMU + magnetometer	IMU + magnetometer	IMU + magnetometer	Set of 4 custom-made anemometers using SDP810 sensors	ultrasonic anemometer FT 205 EV
Wind sensor directional accuracy [°]	±2° within ±10° datum and ±4° beyond ±10° datum	±3°	Not given	Out of scope to discuss	Out of scope to discuss	Not given	±5°
Wind sensor speed accuracy [m/s]	±0.5 m/s V < 15 m/s ±4% V > 15 m/s.	0.30 m/s or <3%, whichever is larger	Not given	Out of scope to discuss	Out of scope to discuss	Not given	0–16 m/s: ±0.3 m/s, 16–40 m/s: ±2% 40–50 m/s: ±4%
Speed bias [m/s]	0.5 m/s	*0.5 m/s 0–0.14 m/s–measurements bias	*0.5 m/s −0.9–+0.17 m/s–measurements bias	Not given	Not given	0.26 m/s	1.5 m/s
Speed RMSE [m/s]–bias not removed	0.6 m/s	Not given	Not given	0.6–1.09 depend on moving average time	0.7 m/s	0.31 m/s (while hovering)	0.5 m/s (1 min average)
Direction bias [°]	−9°	−6°–+22°	−20°–+0°	Not given	Not given	1.73°	−12.52°
Direction RMSE [°]–bias not removed	12°	Not given	Not given	14.02°–29.12° depend on moving average time	7.7°	2.20° (while hovering)	5.33° (1 min average)
Reference sensor	Wind tower sensors on 40 and 55 m	3D sonic anemometer (Gill WindMaster) on a 10-m tower	3D sonic anemometer (Gill WindMaster) on a 10-m tower	Ultrasonic anemometer Young 81,000;	Ultrasonic anemometer, height not given. 5 m apart.	Wind tower, 10m from the ground	Vantage Vue weather station
Ref.	[1]	[2]	[2]	[3]	[4]	[19]	This paper

* Due to downwash–corrected for in the measurements.

## Data Availability

The data is available on demand.
